# Supplementary material for: A conserved morphogenetic mechanism for epidermal ensheathment of nociceptive sensory neurites
Source: eLife. 2019 Mar 11;8:e42455. doi: 10.7554/eLife.42455 (PMC6450671; doi:10.7554/eLife.42455)
Supplement: Supplementary file 1. — Related to Figure 1. [file elife-42455-supp1.docx]

| **Table S1. Related to Figure 1.** Screen for markers associated with epidermal ensheathment channels. | | | | | |
| --- | --- | --- | --- | --- | --- |
| **Membrane markers** | **Epidermal localization** | | **Sheath enriched?** | **Source** |  |
| *UAS-CD4-tdGFP* | Plasma membrane, intracellular membranes | | No | BDSC:35836 |  |
| *UAS-mCD8-GFP* | Plasma membrane, intracellular membranes | | No | BDSC:5137 |  |
| *UAS-myr-GFP* | Plasma membrane, intracellular membranes | | Yes | BDSC:32198 |  |
| *UAS-Asap-GFP* | Plasma membrane, punctate intracellular | | No | BDSC:65849 |  |
| *UAS-Dl-GFP* | Punctate/vesicular | | No | BDSC:8610 |  |
| *UAS-CG10702-GFP* | Punctate/vesicular | | No | BDSC:65857 |  |
| *UAS-Dlg5-GFP* | Punctate/vesicular | | No | BDSC:30928 |  |
| *SdcGFP exon trap* | Plasma membrane, junctional domains | | Yes | Kyoto:115306 |  |
| **Phosphoinositde markers/Lipid binding proteins** | | | | |  |
| *UAS-2xOsh2PH-GFP* (PI4P, PIP2 sensor) | | Plasma membrane, junctional domains | Yes | BDSC:57353 |  |
| *UAS-PLCδ-PH-GFP –* (PIP2 sensor) | | Plasma membrane, junctional domains | Yes | BDSC:39693 |  |
| *UAS-PLCD1-PH-Cerulean –* (PIP2 sensor) | | Plasma membrane, junctional domains | Yes | BDSC:30895 |  |
| *UAS-PLCδ-PH-GFP (S39R)* – point mutant that abrogrates PIP2 binding | | Diffuse cytoplasmic | No | BDSC:39694 |  |
| *UAS-step-GFP* (PIP3 sensor) | | Punctate cytoplasmic, enriched at junctional domains | Weakly concentrated along short stretches | BDSC:65862 |  |
| *UAS-GFP-myc-2xFYVE* (PIP3 sensor) | | Punctate/vesicular and nuclear | No | BDSC:42712 |  |
| **Endocytic markers/effectors** | | | | |  |
| *UAS-Arf51F-GFP* | | Punctate, enriched at junctions | Yes | BDSC:65867 |  |
| *UAS-Arf79F-GFP* | | Punctate/vesicular | No | BDSC:65850 |  |
| *UAS-Arf102F-GFP* | | Punctate/vesicular | No | BDSC:65866 |  |
| *UAS-Arl4-GFP* | | Diffuse | No | BDSC:65868 |  |
| *UAS-Clc-GFP* | | Punctate/vesicular | No | BDSC:7109 |  |
| *UAS-gamma-cop-GFP* | | Punctate/vesicular | No, excluded | BDSC:29711 |  |
| *UAS-YFP-Rab4* | | Punctate/vesicular | No | BDSC:9767 |  |
| *UAS-YFP-Rab-5* | | Punctate/vesicular | No | BDSC:24616 |  |
| *UAS-GFP-Rab-7* | | Punctate/vesicular | No | BDSC:42705 |  |
| *UAS-YFP-Rab11* | | Punctate/vesicular | No | BDSC:50782 |  |
| *UAS-YFP-Rab-21* | | Punctate/vesicular | No | BDSC:23242 |  |
| *UAS-YFP-Rab35* | | Punctate/vesicular | Occasional overlap | BDSC:9821 |  |
| *UAS-spin-RFP* | | Punctate/vesicular | No | BDSC:42716 |  |
| *UAS-Shrub-GFP* | | Punctate/vesicular | No | BDSC:32559 |  |
| **Membrane Tubulation** | | | | |  |
| *UAS-dia-GFP* | | Diffuse | No | BDSC:56751 |  |
| *UAS-EndoA-GFP* | | Punctate | No | this study |  |
| *UAS-EndoB-GFP* | | Punctate, apical | No | this study |  |
| *UAS-Cip4-GFP* | | Punctate | No | (Fricke et al, 2004) |  |
| **Cell-Cell Junctions** | | | | |  |
| *Cora* (antibody stain) | | Junction associated | Yes | DSHB c566.9 |  |
| *UAS-Arm-GFP* | | Junction associated, nuclear | Yes | BDSC:58725 |  |
| *UAS-shg-GFP* | | Junction associated | Yes | BDSC:58445 |  |
| *UAS-baz-GFP* | | Junction associated | Yes, limited | BDSC:65845 |  |
| *UAS-Dlg-GFP* | | Junction associated | No | BDSC:8610 |  |
| *ed^GFP^* exon trap | | Junction-associated | No | Kyoto:115114 |  |
| *Nrg-GFP* | | Junction associated | Yes | BDSC:6844 |  |
| *NrxIV-GFP* | | Junction associated | Yes, weakly | BDSC:50798 |  |
| **Cytoskeleton-related Proteins** | | | | |  |
| *UAS-actin-GFP* | | Diffuse | Yes | BDSC:9258 |  |
| *UAS-GMA-GFP* | | Filamentous network | Yes | BDSC:31176 |  |
| *UAS-APC2-GFP* | | Filamentous network | Labels filaments that align along dendrites | BDSC:8815 |  |
| *UAS-LifeAct.mGFP* | | Filamentous network | Yes | BDSC:58717 |  |
| *UAS-Arp3-GFP* | | Diffuse | No | BDSC:39722 |  |
| *UAS-Arpc1-GFP* | | Diffuse | No | BDSC:26692 |  |
| *UAS-capu-GFP* | | Cytoplasmic/punctate | No | BDSC:24764 |  |
| *UAS-dpod1-GFP* | | Membrane ruffles | No | BDSC:8800 |  |
| *UAS-Pak-GFP* | | Diffuse | No | BDSC:52299 |  |
| *UAS-Rho1-GFP* | | Diffuse, enriched at cell junctions | Yes | BDSC:9393 |  |
| *UAS-Rok-GFP* | | Diffuse | No | BDSC:52290 |  |
| *UAS-shot-GFP* | | Diffuse | No | BDSC:29044 |  |
| *UAS-GFP-blr* | | Apical membranes | No | BDSC:8659 |  |
| *UAS-spir-GFP* | | Diffuse | No | BDSC:8820 |  |
| *UAS-GFP-sstn* | | Junctional domains, nuclei | No | BDSC:65863 |  |
| *UAS-alpha-tubulin84B-GFP* | | Filamentous network | No | BDSC:7373 |  |
| *UAS-EB1-GFP* | | Cytoplasmic puncta | No | BDSC:35512 |  |
| *UAS-hook-GFP* | | Diffuse cytoplasmic | No | BDSC:65858 |  |
| *UAS-jar-GFP* | | Diffuse cytoplasmic | No | BDSC:67606 |  |
| *UAS-Khc-GFP* | | Filamentous network | No | BDSC:9648 |  |
| *UAS-GFP-Myo10A* | | Large cytoplasmic puncta | No | BDSC:24781 |  |
| *UAS-GFP-Myo31DF* | | Filamentous network | No | BDSC:1521 |  |
| *UAS-GFP-NinaC* | | Diffuse | No | BDSC:43347 |  |
| *UAS-GFP-DCTN1-p150* | | Diffuse | No | BDSC:29982 |  |
| *UAS-Unc104-GFP* | | Filamentous network | No | BDSC:24786 |  |
| *UAS-Supervillin-GFP* | | Diffuse | No | BDSC:66165 |  |
| *UAS-GFP-RhoGAP19D* | | Diffuse | No | BDSC:66167 |  |
| **Polarity** | | | | |  |
| *UAS-Par6-GFP* | | Diffuse; enriched at junctional domains and in nucleus | No | BDSC:65847 |  |
| *UAS-fy-GFP* | | Nuclear, diffuse cytoplasmic | No | BDSC:66513 |  |
| *UAS-sds22.GFP* | | Diffuse, nuclear | No | BDSC:65851 |  |
| **Organelle markers** | | | | |  |
| *UAS-EGFP (cytosolic)* | | Diffuse | No | BDSC:5431 |  |
| *UAS-mito-GFP* | | Mitochondria-associated | No | BDSC:8443 |  |
| *UAS-GFP-Golgi* | | Punctate | No | BDSC31422 |  |
| *UAS-GFP-KDEL* | | Punctate | No | BDSC:9898 |  |
| *UAS-GFP.SKL* | | Punctate | No | BDSC:28881 |  |
| *UAS-eGFP-Atg5* | | Diffuse | No | BDSC:59848 |  |
| *UAS-Atg8-GFP* | | Diffuse | No | BDSC:52005 |  |
| *UAS-GFP-LAMP* | | Punctate | No | BDSC:42714 |  |
| **Signaling/Other** | | | | |  |
| *UAS-Aplip-GFP* | | Diffuse | No | BDSC:24634 |  |
| *UAS-hiw-GFP* | | Diffuse cytosolic | No | BDSC:51640 |  |
| *UAS-src-EGFP* | | Diffuse cytosolic | No | BDSC:5429 |  |
| *UAS-bsk-GFP* | | Diffuse | No | BDSC:59267 |  |
| *UAS-Dronc-GFP* | | Diffuse cytosolic, concentrated in the nucleus | No | BDSC:57659 |  |
| *UAS-Myc-fry-GFP* | | Punctate cytosolic | No | BDSC:32106 |  |
| *UAS-Fak-GFP* | | Punctate; some enrichment at junctional domains | No | (Grabbe *et al.*, 2004) |  |
| *UAS-muskelin-GFP* | | Diffuse cytosolic | No | BDSC:65860 |  |
| *UAS-roc2-GFP* | | Diffuse cytosolic, concentrated in the nucleus | No | BDSC:65861 |  |

BDSC, Bloomington Drosophila Stock Center; Kyoto, Kyoto Drosophila Stock Center
