# Supplementary material for: A conserved morphogenetic mechanism for epidermal ensheathment of nociceptive sensory neurites
Source: eLife. 2019 Mar 11;8:e42455. doi: 10.7554/eLife.42455 (PMC6450671; doi:10.7554/eLife.42455)
Supplement: Supplementary file 2. — Related to Figures 1–7 and supplements. [file elife-42455-supp2.docx]

| **Table S2. Related to Figures 1-7 and supplements.** Experimental *Drosophila* and zebrafish genotypes used in this study. | | |
| --- | --- | --- |
| **Figure** | **Genotype** |  |
| Figure 1B-1C | *w^1118^* |  |
| Figure 1D-1E | *w^1118^; UAS-CD4-tdGFP/ppk-CD4-tdTomato ; A58-Gal4, ppk-CD4-tdTomato/+* |  |
| Figure 1F-1K | *w^1118^; ppk-CD4-tdTomato/+ ; A58-Gal4, ppk-CD4-tdTomato/UAS-PLCγ-PH-GFP* |  |
| Figure 1L-1M | *w^1118^; ppk-CD4-tdTomato/+ ; A58-Gal4, ppk-CD4-tdTomato/UAS-dArf6-GFP* |  |
| Figure 1N-1O | *w^1118^; ppk-CD4-tdTomato/+ ; A58-Gal4, ppk-CD4-tdTomato/UAS-Rho1-GFP* |  |
| Figure 1P-1Q | *w^1118^; ppk-CD4-tdTomato/+ ; A58-Gal4, ppk-CD4-tdTomato/UAS-UAS-GMA-GFP* |  |
| Figure 1R-1S | *w^1118^; ppk-CD4-tdTomato/+ ; A58-Gal4, ppk-CD4-tdTomato/+* |  |
| Figure 1T-1U | *w^1118^; ppk-CD4-tdTomato/+ ; A58-Gal4, ppk-CD4-tdTomato/UAS-shg-GFP* |  |
| Figure 1-S1B | *w^1118^;; A58-Gal4/UAS-PLCδ-PH-GFP* |  |
| Figure 1-S1C | *w^1118^; ppk-CD4-tdTomato/+ ; A58-Gal4, ppk-CD4-tdTomato/UAS-PLCδ-PH-PH^S39R^-GFP* |  |
| Figure 1-S1D | *w^1118^; ppk-CD4-tdTomato/+ ; A58-Gal4, ppk-CD4-tdTomato/UAS-PLCδ-PH-GFP* |  |
| Figure 1-S1E | *w^1118^; ppk-CD4-tdTomato/+ ; A58-Gal4, ppk-CD4-tdTomato/UAS-2xOsh2PH-GFP* |  |
| Figure 1-S1F | *w^1118^; ppk-CD4-tdTomato/+ ; ppk-CD4-tdTomato/NrxIV^GFP^* |  |
| Figure 1-S2 | *w^1118^; ppk-CD4-tdTomato/+ ; A58-Gal4, ppk-CD4-tdTomato/UAS-PLCδ-PH-GFP* |  |
| Figure 2B-2C | *PIP2 [TgBAC(tp63:GAL4FF); Tg(UAS:GFP-PH-PLC)]* |  |
| Figure 2D-2E | *F-actin [TgBAC(tp63:GAL4FF); Tg(UAS:lifeact-GFP)]* |  |
| Figure 2F-2G | *α-catenin [Gt(ctnna-citrine)]* |  |
| Figure 2H | *PIP2 [TgBAC(tp63:GAL4FF); Tg(UAS:GFP-PH-PLC)], trigeminal axons [Tg(isl1[ss]:LEXA-VP16,LEXAop:tdTomato)]* |  |
| Figure 2I | *α-catenin [Gt(ctnna-citrine)], trigeminal axons [Tg(isl1:GAL4-VP16,UAS:RFP)]* |  |
| Figure 2J-2K | *PIP2 [TgBAC(tp63:GAL4FF); Tg(UAS:EGFP-PH-PLC)], lateral line axons [Tg(neurod:mTangerine) in transient]* |  |
| Figure 2-S1A-S1C | *axons [Tg(isl1:GAL4-VP16,UAS:RFP)], GFP-CAAX [Tg(krtt1c19e:GFP-CAAX) in transient]* |  |
| Figure 2-S1D-S1F | *axons [Tg(isl1:GAL4-VP16,UAS:EGFP)], E-cadherin [Gt(cdh1-tdtomato)]* |  |
| Figure 2-S1G-S1I | *axons [Tg(isl1:GAL4-VP16,UAS:RFP)], Dscl2 [TgBAC(dscl2:Dscl2-GFP) in transient]* |  |
| Figure 2-S1J-S1L | *axons [Tg(isl1:GAL4-VP16,UAS:RFP)], Dspa [TgBAC(dspa:Dspa-GFP) in transient]* |  |
| Figure 2-S1M-S1O | *axons [Tg(isl1:GAL4-VP16,UAS:RFP)], Jupa [Gt(jupa-citrine)]* |  |
| Figure 3A-3C | *w^1118^; elav-lexA, lexAOP-CD4-tdTomato/+ ; A58-Gal4, UAS-PLCδ-PH-GFP, ppk-CD4-tdTomato/+* |  |
| Figure 3D-3F | *w^1118^; lexAOP-CD4-tdTomato/+ ; A58-Gal4, UAS-PLCδ-PH-GFP, ppk-CD4-tdTomato/NompC-lexA* |  |
| Figure 3F | *w^1118^; lexAOP-CD4-tdTomato/+ ; A58-Gal4, UAS-PLCδ-PH-GFP/NompC-lexA* |  |
| Figure 3G-3J | *PIP2 [TgBAC(tp63:GAL4FF); Tg(UAS:GFP-PH-PLC)], trigeminal axons [Tg(isl1[ss]:LEXA-VP16,LEXAop:tdTomato) in transient]* |  |
| Figure 3-S1A-S1C | *w^1118^; 98b-Gal4/+; UAS-mCD4-tdGFP/+* |  |
| Figure 3-S1D-S1F | *w^1118^; Gal4^GMR37B02^/+; UAS-mCD4-tdGFP/+* |  |
| Figure 4A-4H | *w^1118^; ppk-CD4-tdTomato/+ ; A58-Gal4, ppk-CD4-tdTomato/UAS-PLCδ-PH-GFP* |  |
| Figure 4I-4K | *w^1118^; ppk-Gal4/UAS-rpr ; ppk-Gal4/+* |  |
| Figure 4L-4N | *w^1118^; 21-7-Gal4, UAS-mCD8-GFP/+* |  |
| Figure 4O-4T | *w^1118^; elav-lexA, lexAOP-CD4-tdTomato/+ ; A58-Gal4, UAS-PLCδ-PH-GFP, ppk-CD4-tdTomato/+* |  |
| Figure 4U | *PIP2 [TgBAC(tp63:GAL4FF); Tg(UAS:GFP-PH-PLC)]* |  |
| Figure 4V | *F-actin [TgBAC(tp63:GAL4FF); Tg(UAS:lifeact-GFP)]* |  |
| Figure 4W | *α-catenin [Gt(ctnna-citrine)]* |  |
| Figure 4X | *PIP2 [TgBAC(tp63:GAL4FF); Tg(UAS:GFP-PH-PLC)]* |  |
| Figure 4Y | *F-actin [TgBAC(tp63:GAL4FF); Tg(UAS:lifeact-GFP)]* |  |
| Figure 4Z | *α-catenin [Gt(ctnna-citrine)]* |  |
| Figure 4AA | *PIP2 [TgBAC(tp63:GAL4FF); Tg(UAS:GFP-PH-PLC)]; trigeminal axons [Tg(isl1[ss]:LEXA-VP16,LEXAop:tdTomato)]* |  |
| Figure 4-S1 | *w^1118^; ppk-CD4-tdTomato/+ ; A58-Gal4, ppk-CD4-tdTomato/UAS-PLCδ-PH-GFP* |  |
| Figure 4-S2 | *w^1118^; ; ppk-CD4-tdTomato/ppk-CD4-tdTomato* |  |
| Figure 4-S3 | *w^1118^; ppk-CD4-tdTomato/+ ; A58-Gal4, ppk-CD4-tdTomato/UAS-PLCδ-PH-GFP* |  |
| Figure 5A-5B | *w^1118^; ppk-CD4-tdTomato/+ ; A58-Gal4, UAS-dArf6-GFP/UAS-PLCδ-PH-Cerulean* |  |
| Figure 5C-5D | *w^1118^; ppk-CD4-tdTomato/+ ; A58-Gal4, UAS-GMA-GFP/UAS-PLCδ-PH-Cerulean* |  |
| Figure 5E-5F | *w^1118^; ppk-CD4-tdTomato/+ ; A58-Gal4, UAS-GFP-cora^1-383^/UAS-PLCδ-PH-Cerulean* |  |
| Figure 5G | *PIP2 [TgBAC(tp63:GAL4FF); Tg(UAS:GFP-PH-PLC)], GFP-CAAX [TgBAC(tp63:GAL4FF); Tg(UAS:GFP-CAAX)], F-actin [TgBAC(tp63:GAL4FF); Tg(UAS:lifeact-GFP)], Jupa [Gt(jupa-citrine)], α-catenin [Gt(ctnna-citrine)]* |  |
| Figure 5H | *See details for Figure 5-S3* |  |
| Figure 5-S1A | *w^1118^; ppk-CD4-tdTomato/+ ; A58-Gal4, UAS-GMA-GFP/UAS-PLCδ-PH-Cerulean* |  |
| Figure 5-S1B | *w^1118^; ppk-CD4-tdTomato/+ ; A58-Gal4, UAS-dArf6-GFP/UAS-PLCδ-PH-Cerulean* |  |
| Figure 5-S1C | *w^1118^; ppk-CD4-tdTomato/+ ; A58-Gal4, UAS-GFP-cora^1-383^/+* |  |
| Figure 5-S2 | *PIP2 [TgBAC(tp63:GAL4FF); Tg(UAS:GFP-PH-PLC)], TG axons [Tg(isl1[ss]:LEXA-VP16,LEXAop:tdTomato)]* |  |
| Figure 5-S3A-B | *w^1118^; ppk-CD4-tdTomato/+ ; A58-Gal4, UAS-PLCδ-PH-GFP, ppk-CD4-tdTomato/+* |  |
| Figure 5-S3C-D | *w^1118^; ppk-CD4-tdTomato/UAS-PI4K(RNAi) ; A58-Gal4, UAS-PLCδ-PH-GFP, ppk-CD4-tdTomato/+* |  |
| Figure 5-S3E-F | *w^1118^; ppk-CD4-tdTomato/+ ; A58-Gal4, UAS-PLCδ-PH-GFP, ppk-CD4-tdTomato/+* |  |
| Figure 5-S3G-H | *w^1118^; ppk-CD4-tdTomato/UAS-shi^DN^ ; A58-Gal4, UAS-PLCδ-PH-GFP, ppk-CD4-tdTomato/+* |  |
| Figure 5-S3I-J | *w^1118^; ppk-CD4-tdTomato/UAS-cora(RNAi) ; A58-Gal4, ppk-CD4-tdTomato/+* |  |
| Figure 5-S3K-L | *w^1118^; ppk-CD4-tdTomato/UAS-PI4K(RNAi) ; A58-Gal4, ppk-CD4-tdTomato/+* |  |
| Figure 5-S3M-N | *w^1118^; ppk-CD4-tdTomato/UAS-shi^DN^ ; A58-Gal4, ppk-CD4-tdTomato/+* |  |
| Figure 5-S3O-P | *w^1118^; ppk-CD4-tdTomato/UAS-cora(RNAi) ; A58-Gal4, ppk-CD4-tdTomato/+* |  |
| Figure 6A | *w^1118^*, *ppk-mCD8-GFP; ; A58-Gal4, cha-gal80/+* |  |
| Figure 6B | *w^1118^*, *ppk-mCD8-GFP; UAS-PI4K(RNAi)/+ ; A58-Gal4, cha-gal80/+* |  |
| Figure 6C | *w^1118^*, *ppk-mCD8-GFP; UAS-shi^DN^/+ ; A58-Gal4, cha-gal804/+* |  |
| Figure 6D | *w^1118^*, *ppk-mCD8-GFP; UAS-shi^ts^/+ ; A58-Gal4, cha-gal80/+* |  |
| Figure 6E | *w^1118^*, *ppk-mCD8-GFP; UAS-cora(RNAi)/+ ; A58-Gal4, cha-gal80/+* |  |
| Figure 6F | *w^1118^*, *ppk-mCD8-GFP; UAS-shg(RNAi)/+ ; A58-Gal4, cha-gal80/+* |  |
| Figure 6I | *w^1118^*, *ppk-mCD8-GFP; ; A58-Gal4, cha-gal80/+* |  |
| Figure 6J | *w^1118^*, *ppk-mCD8-GFP; UAS-PI4K(RNAi)/+ ; A58-Gal4, cha-gal80/+* |  |
| Figure 6K | *w^1118^*, *ppk-mCD8-GFP; UAS-cora(RNAi)/+ ; A58-Gal4, cha-gal80/+* |  |
| Figure 6N-6Q | *w^1118^; ppk-CD4-tdTomato/+ ; A58-Gal4, ppk-CD4-tdTomato/UAS-PLCδ-PH-GFP* |  |
| Figure 6R | *w^1118^*, *ppk-mCD8-GFP; ; A58-Gal4, cha-gal80/+* |  |
| Figure 6S | *w^1118^*, *ppk-mCD8-GFP; UAS-PI4K(RNAi)/+ ; A58-Gal4, cha-gal80/+* |  |
| Figure 6T | *w^1118^*, *ppk-mCD8-GFP; UAS-cora(RNAi)/+ ; A58-Gal4, cha-gal80/+* |  |
| Figure 6-S1A | *w^1118^; ppk-CD4-tdTomato/+ ; A58-Gal4, ppk-CD4-tdTomato/+* |  |
| Figure 6-S1B | *w^1118^; ppk-CD4-tdTomato/+ ; A58-Gal4 UAS-PLCδ-PH-GFP, ppk-CD4-tdTomato/UAS-PLCδ-PH-GFP (2 copies PLCδ-PH-GFP)* |  |
| Figure 6-S1C-S1D | *w^1118^; ppk-CD4-tdTomato/+ ; A58-Gal4, ppk-CD4-tdTomato/UAS-PLCδ-PH-GFP (1 copy PLCδ-PH-GFP)* |  |
| Figure 6-S2 | *w^1118^; ppk-CD4-tdTomato/+ ; A58-Gal4, ppk-CD4-tdTomato/UAS-PLCδ-PH-GFP* |  |
| Figure 6-S3 | *w^1118^; ppk-CD4-tdTomato/+ ; A58-Gal4 UAS-PLCδ-PH-GFP, ppk-CD4-tdTomato/+* |  |
| Figure 7A | *w^1118^; ; A58-Gal4, cha-gal80/+* (control) |  |
| Figure 7A | *w^1118^; +; ppk-Gal4/UAS-KIR2.1* (*C4da>Kir*) |  |
| Figure 7A | *w^1118^; +; ppk-Gal4/+ ; UAS-mys, UAS-mew/+* (*C4da>Integrin*s) |  |
| Figure 7A | *w^1118^; ; A58-Gal4 bantam^Δ1^*/*bantam^Δ1^ (bantam^Δ1^)* |  |
| Figure 7A | *w^1118^; UAS-PI4K(RNAi) ; A58-Gal4, cha-gal80/+* (*Epi>PI4K-RNAi*) |  |
| Figure 7A | *w^1118^; ; A58-Gal4, cha-gal80/UAS-PIS(RNAi)* (*Epi>PISRNAi*) |  |
| Figure 7A | *w^1118^; ; A58-Gal4, cha-gal80/+* (PBP10 feeding) |  |
| Figure 7A | *w^1118^; ; A58-Gal4 bantam^Δ1^*/*bantam^Δ1^* (*bantam^Δ1^*+ PBP10) |  |
| Figure 7A | *w^1118^; UAS-shi^DN^ ; A58-Gal4, cha-gal80/+* (*Epi>shiDN*) |  |
| Figure 7A | *w^1118^; UAS-cora(RNAi) ; A58-Gal4, cha-gal80/+* (*Epi>coraRNAi*) |  |
| Figure 7B-7C | *w^1118^; ; A58-Gal4, cha-gal80/+* (control) |  |
| Figure 7B-7C | *w^1118^; UAS-PI4K(RNAi) ; A58-Gal4, cha-gal80/+* (*Epi>PI4K-RNAi*) |  |
| Figure 7B-7C | *w^1118^; UAS-shi^DN^ ; A58-Gal4, cha-gal80/+* (*Epi>shiDN*) |  |
| Figure 7B-7C | *w^1118^; UAS-cora(RNAi) ; A58-Gal4, cha-gal80/+* (*Epi>coraRNAi*) |  |
| Figure 7-S1 | *w^1118^; ; A58-Gal4, cha-gal80/+* |  |
